# Supplementary material for: Variability in locomotor dynamics reveals the critical role of feedback in task control
Source: eLife. 2020 Jan 23;9:e51219. doi: 10.7554/eLife.51219 (PMC7041942; doi:10.7554/eLife.51219)
Supplement: Transparent reporting form [file elife-51219-transrepform.pdf]

eLIFE
1st Floor
24 Hills Road
Cambridge CB2 1JP, UK
P 01223 855340
W elifesciences.org
T @elife

eLife's transparent reporting form

We encourage authors to provide detailed information *within their submission* to facilitate the interpretation and replication of experiments. Authors can upload supporting documentation to indicate the use of appropriate reporting guidelines for health-related research (see EQUATOR Network), life science research (see the BioSharing Information Resource), or the ARRIVE guidelines for reporting work involving animal research. Where applicable, authors should refer to any relevant reporting standards documents in this form.

If you have any questions, please consult our Journal Policies and/or contact us: editorial@elifesciences.org.

Sample-size estimation

- You should state whether an appropriate sample size was computed when the study was being designed
- You should state the statistical method of sample size computation and any required assumptions
- If no explicit power analysis was used, you should describe how you decided what sample (replicate) size (number) to use

Please outline where this information can be found within the submission (e.g., sections or figure legends), or explain why this information doesn't apply to your submission:

Our analysis is mainly based on three measurements for each trial: movement of the refuge (input), movement of the fish (output), and movement of the nodal point (control signal). The movements of the refuge and fish were digitized using custom image tracking software implemented in Matlab. The nodal point position, however, was hand clicked for each frame: we made 18000 nodal point measurements in 106 video segments from three fish. Three fish was the minimum necessary to implement virtual brain transplants, namely computationally swapping controllers and plants across individuals. The details of the sample sizes and handling of data are described in the Materials and Methods.

Replicates

- You should report how often each experiment was performed
- You should include a definition of biological versus technical replication
- The data obtained should be provided and sufficient information should be provided to indicate the number of independent biological and/or technical replicates
- If you encountered any outliers, you should describe how these were handled
- Criteria for exclusion/inclusion of data should be clearly stated
- High-throughput sequence data should be uploaded before submission, with a private link for reviewers provided (these are available from both GEO and ArrayExpress)

Please outline where this information can be found within the submission (e.g., sections or figure legends), or explain why this information doesn't apply to your submission:

eLife Sciences Publications, Ltd is a limited liability non-profit non-stock corporation incorporated in the State of Delaware, USA, with company number 5030732, and is registered in the UK with company number FC030576 and branch number BR015634 at the address 1st Floor, 24 Hills Road, Cambridge CB2 1JP | August 2014

We collected 3-5 trials for each of three test frequencies of refuge movements per individual. The trials were randomized across frequencies and separated by a minimum interval of two minutes.

We extracted segments with durations of 3 to 10 seconds from each trial for analysis. We used 11-16 segments for each experimental condition per individual. This refers to technical replicates (replications within each individual) used in our study. This analysis was repeated for three individuals, which refers to biological replicates (replications across individuals).

We included all valid replicates in our analysis: no replicates were eliminated. We did not exclude any "outlier" data.

Because the study concerns counter-propogating waves of the ribbon fin (which generates the nodal point), recordings in which the animal was not using counter-propogating waves were excluded. For example, fish routinely use unidirectional waves for locomotion - these data were not analyzed in the same way that a study on trotting in horses would exclude data collected when the horse was galloping. The details about replication can be found in the Materials and Methods.

Statistical reporting

- Statistical analysis methods should be described and justified
- Raw data should be presented in figures whenever informative to do so (typically when N per group is less than 10)
- For each experiment, you should identify the statistical tests used, exact values of N, definitions of center, methods of multiple test correction, and dispersion and precision measures (e.g., mean, median, SD, SEM, confidence intervals; and, for the major substantive results, a measure of effect size (e.g., Pearson's r, Cohen's d)
- Report exact p-values wherever possible alongside the summary statistics and 95% confidence intervals. These should be reported for all key questions and not only when the p-value is less than 0.05.

Please outline where this information can be found within the submission (e.g., sections or figure legends), or explain why this information doesn't apply to your submission:

We used bootstrapping analysis while estimating the frequency response functions (FRFs) of the plant dynamics. We used the bootstrapped estimates to find the mean FRFs at each frequency due to our limited data. It was also useful to see the distribution of FRFs around the mean. This helped us to interpret the structure of the parametric transfer functions and uncertainty bounds across frequencies. The details for the bootstrapping analysis for estimating the FRFs are explained in Materials and Methods.

We used a similar approach while quantifying the trial-to-trial variability across individuals. Note that we performed constant frequency experiments at different frequencies. We made 37 observations of FRFs at $f = 0.55Hz$, 35 observations at $f = 0.95Hz$, and 34 at $f = 2.05Hz$. To estimate the trial-to-trial variability at the frequencies that were not explicitly tested, we used a parametric approach. Specifically, we constructed 1000 triplets by randomly selecting one FRF from the set of each test frequency. We estimated a

eLife Sciences Publications, Ltd is a limited liability non-profit non-stock corporation incorporated in the State of Delaware, USA, with company number 5030732, and is registered in the UK with company number FC030576 and branch number BR015634 at the address 1st Floor, 24 Hills Road, Cambridge CB2 1JP | August 2014

parametric transfer function for each of these 1000 triplets and computed the range of gain and phase responses as the trial-to-trial variability across fish. This statistical approach was useful for us to combine FRFs from different frequencies. The details for these computations are also presented in Materials and Methods.

We did not analyze the effects of experimental parameters on the results. Thus, we do not report any significance analysis in the manuscript.

(For large datasets, or papers with a very large number of statistical tests, you may upload a single table file with tests, Ns, etc., with reference to sections in the manuscript.)

Group allocation

- Indicate how samples were allocated into experimental groups (in the case of clinical studies, please specify allocation to treatment method); if randomization was used, please also state if restricted randomization was applied
- Indicate if masking was used during group allocation, data collection and/or data analysis

Please outline where this information can be found within the submission (e.g., sections or figure legends), or explain why this information doesn't apply to your submission:

The experimental trials per individual was randomized across frequencies of refuge movement to prevent adaptation to specific constant frequency stimulus.

Additional data files ("source data")

- We encourage you to upload relevant additional data files, such as numerical data that are represented as a graph in a figure, or as a summary table
- Where provided, these should be in the most useful format, and they can be uploaded as "Source data" files linked to a main figure or table
- Include model definition files including the full list of parameters used
- Include code used for data analysis (e.g., R, MatLab)
- Avoid stating that data files are "available upon request"

Please indicate the figures or tables for which source data files have been provided:

An archived version of the dataset and analysis codes will be made available through the Johns Hopkins University Data Archive through DOI: 10.7281/T1/UDTJPD. The data archive can be cited as:

Uyanik, Ismail; Sefati, Shahin; Stamper, Sarah A.; Cho, Kyoung-A; Ankarali, M. Mert; Fortune, Eric, S.; Cowan, Noah J., 2019, "Data associated with publication "Variability in Locomotor Dynamics Reveals the Critical Role of Feedback in Task Control"", https://doi.org/10.7281/T1/UDTJPD , Johns Hopkins University Data Archive.

In addition, full list of parameters that are obtained through system identification experiments are reported in Table I and Table II in the main manuscript file.

eLife Sciences Publications, Ltd is a limited liability non-profit non-stock corporation incorporated in the State of Delaware, USA, with company number 5030732, and is registered in the UK with company number FC030576 and branch number BR015634 at the address 1st Floor, 24 Hills Road, Cambridge CB2 1JP | August 2014
